# Heliorhodopsin Helps Photolyase to Enhance the DNA Repair Capacity

**DOI:** 10.1128/spectrum.02215-22

**Published:** 2022-10-11

**Authors:** Jin-gon Shim, Shin-Gyu Cho, Se-Hwan Kim, Kimleng Chuon, Seanghun Meas, Ahreum Choi, Kwang-Hwan Jung

**Affiliations:** a Department of Life Science, Sogang University, Seoul, South Korea; b Research Institute for Basic Science, Sogang University, Seoul, South Korea; c Research Center for Endangered Species, National Institute of Ecology, Yeongyang-gun, Gyeongsangbuk-do, South Korea; University at Buffalo, State University of New York

**Keywords:** DNA repair, photolyase, microbial rhodopsin, protein-protein interaction, heliorhodopsin, evolution

## Abstract

Light quality is a significant factor for living organisms that have photosensory systems, such as rhodopsin, a seven alpha-helical transmembrane protein with the retinal chromophore. Here, we report, for the first time, the function of new rhodopsin, which is an inverted 7-transmembrane protein, isolated from Trichococcus flocculiformis*. T. flocculiformis* heliorhodopsin (TfHeR) works as a regulatory helper rhodopsin that binds with class 2 cyclobutane pyrimidine dimer (CPDII) photolyase to broaden the spectrum and upregulate DNA repair activity. We have confirmed their interaction through isothermal titration calorimetry (dissociation constant of 21.7 μM) and identified the charged residues for the interaction. Based on *in vivo* and *in vitro* experiments, we showed that the binding of heliorhodopsin with photolyase improved photolyase activity by about 3-fold to repair UV-caused DNA damage. Also, the DNA repair activity of TfHeR/*T. flocculiformis* photolyase (TfPHR) was observed in the presence of green light. Our results suggested that heliorhodopsin directly controls the activity of photolyase and coevolves to broaden the activity spectrum by protein-protein interaction.

**IMPORTANCE** This study reports a function for Heliorhodopsin working as a regulatory helper rhodopsin that with CPDII photolyase to broaden the spectrum and upregulating the DNA repair activity. Our results suggested that heliorhodopsin directly controls photolyase activity and coevolves to broaden the DNA repair capacity by protein-protein interaction.

## INTRODUCTION

**FIG 1:**
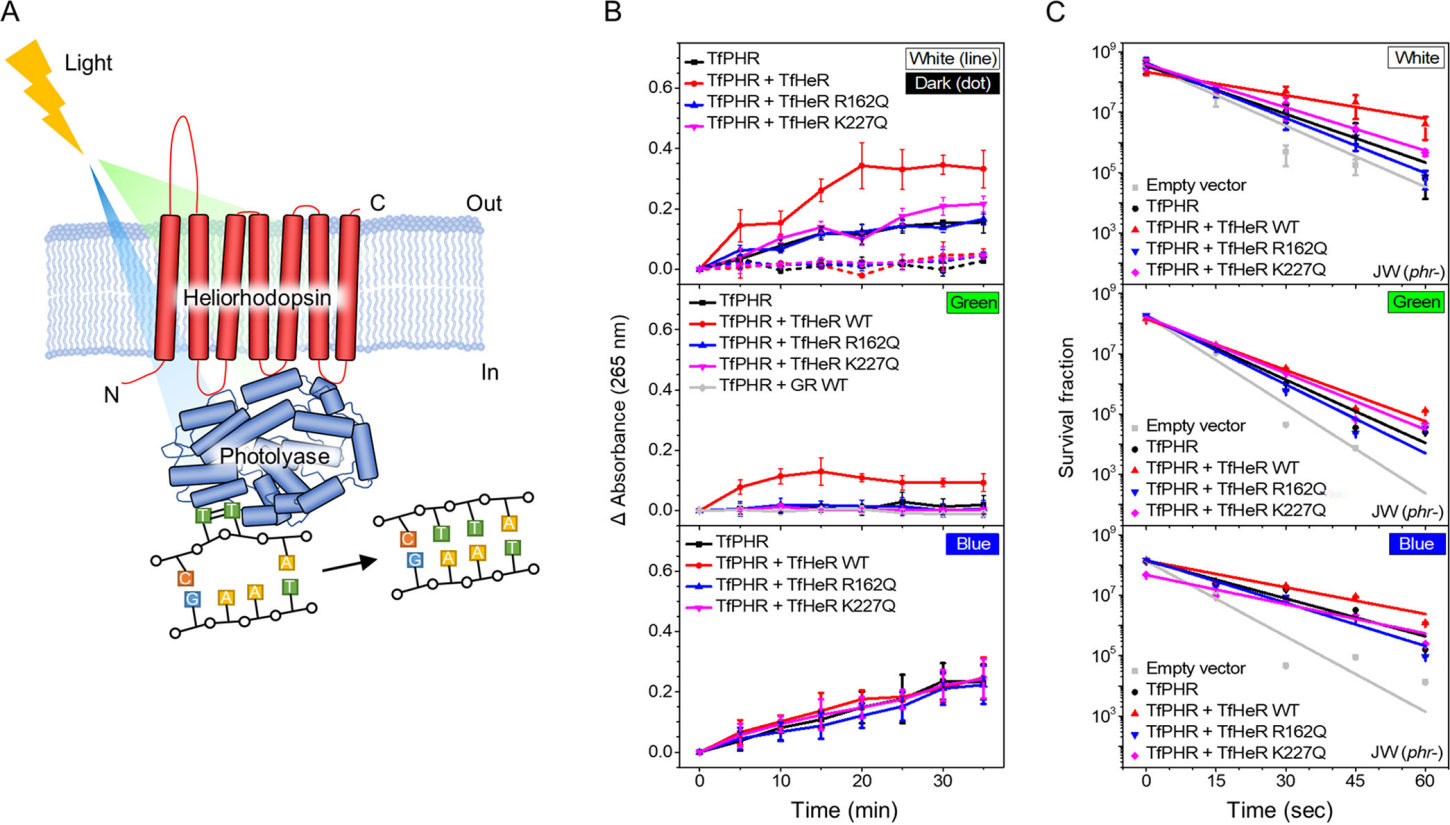
Schematic model and function of TfHeR bound to TfPHR. (A) UV-induced cyclobutane pyrimidine dimers (green squares) repaired via photolyase and heliorhodopsin in the presence of green light. (B) Repair of pyrimidine dimers *in vitro*. The recovery of cyclobutane pyrimidine dimers generated by photolyase measured under white, green, and blue lights. DNA recovery was measured at 265 nm. (C) Test for photolyase activity in the cell. Each experiment was performed in triplicate and a standard deviation was obtained. Each experiment was performed seven times and the standard deviation was calculated. Light color is indicated in the upper right corner of the graphs.

Microbial rhodopsin, which is classified into diverse functional clusters, is abundant in the environment (1). The contribution of microbial rhodopsin, which functions in the presence of light, has been reported in ion transport, ion channel activity, and photo-sensing transduction. Among the ion-pumping rhodopsins, the most abundant cluster belongs to a new type of heliorhodopsin (HeR) with unknown functions, indicating that microbial rhodopsins have a variety of biological functions (2–4). HeRs exhibit an inverse membrane topology that changes direction in the membrane with an N terminus toward the cytoplasm but retains characteristics of microbial rhodopsin in terms of structure and photoactivation response (2). HeR was mainly found and studied in Gram-negative bacteria (5, 6). Bellilinea caldifistulae HeR (BcHeR) extracted from the Gram-negative Eubacterium Bellilinea caldifistulae has been identified and studied (5). *Thermoplasmatales archaeon* HeR (TaHeR) can bind to zinc, and the discovery of various HeRs expands the community (6). Studies on HeR conformational changes and photoisomerization have been reported, but their functions are unknown (7, 8). An evolutionary approach using bioinformatics is required to elucidate the function. Various approaches have been applied to discover the biological function of HeR, and several studies are being conducted for the exploration of the genomic context of HeR (9, 10). In this study, using a helio-opsin gene found in Trichococcus flocculiformis, a Gram-positive bacterium isolated from bulking sludge (11) (Fig. 1A), we studied the biological function of *T. flocculiformis* heliorhodopsin (TfHeR), involving CPDII photolyase, among several candidates located in the same operon where the helio-opsin gene is located. The interaction of TfHeR and photolyase was observed through biophysical and biochemical analysis, and functional relationships were investigated.

## RESULTS AND DISCUSSION

### Sequencing and topological information of TfHeR.

**FIG 2:**
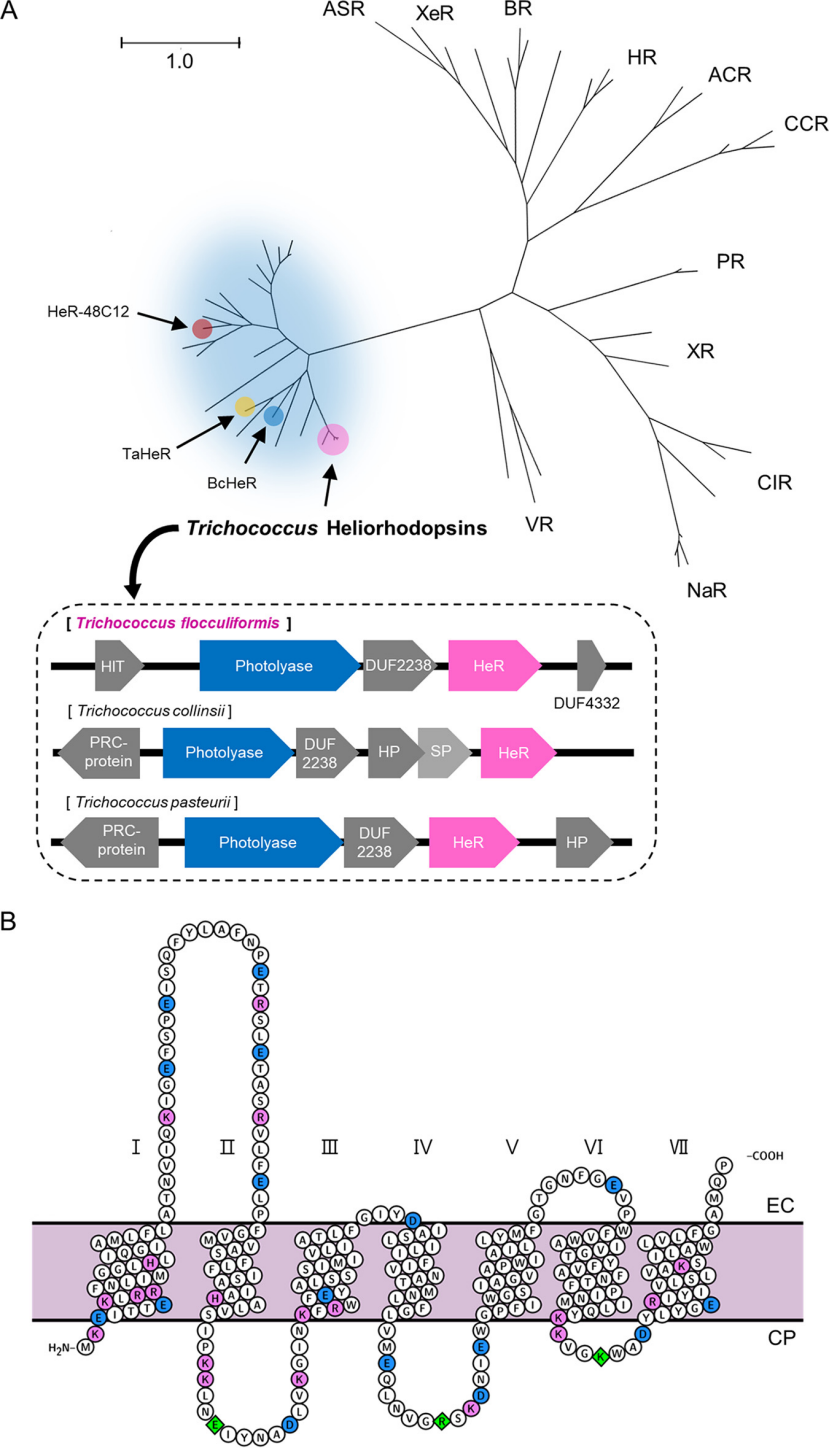
Microbial rhodopsins and TfHeR. (A) The phylogenetic tree of type-1 rhodopsins and heliorhodopsin is represented. Light blue circle indicates heliorhodopsin groups. 48C12, Bellilinea caldifistulae HeR (BcHeR), and *Thermoplasmatales archaeon* HeR (TaHeR) are indicated with a red and blue circle, respectively (5, 6). A group of three *Trichococcus* heliorhodopsins is indicated with a pink circle and each operon of *Trichococcus* species is shown with photolyase, heliorhodopsin, and other genes in the operon. ASR, *Anabaena* sensory rhodopsin; XeR, xenorhodopsin, BR, bacteriorhodopsin; HR, halorhodopsin; ACR, anion channelrhodopsin; CCR, cation channelrhodopsin; PR, proteorhodopsin; XR, xanthorhodopsin; CIR, chloride ion-pumping rhodopsin; NaR, sodium ion-pumping rhodopsin; VR, viral rhodopsin. NCBI reference sequence of Trichococcus species, *T.flocculiformis*, NZ_FOQC01000073.1; *T.collinsii*, NZ_FNQH01000003.1; *T.pasterurii*, NZ_FONM01000005.1. Each protein presented in the operon of *Trichoccous* is a HIT protein. HIT family of proteins; DUF2238, domain of unknown function 2238; DUF4332, domain of unknown function 4332; PRC-protein, pentapeptide repeat-containing protein. HIT, HIT family protein; HP, hypothetical protein; SP, signal peptidase I. (B) The predicted secondary structure of TfHeR. The transmembrane helices are indicated A to F. The structure prediction was based on the crystal structure of 48C12 (23). It was predicted based on the 48C12 structure, and the N-terminal was predicted to be relatively short. Negatively and positively charged residues are highlighted with a pink and blue circle, respectively. Residues related to photolyase binding are highlighted with a green circle.

A helio-opsin gene found in Trichococcus flocculiformis, a Gram-positive bacterium isolated from bulking sludge (11), led to the prediction of inverse seven transmembrane helices and lysine in the seventh helix (Fig. 2B and Fig. S1A). In the genome of Trichococcus flocculiformis, the helio-opsin gene was the only opsin gene, and another type of microbial rhodopsin was not found. Sequence alignment and prediction revealed that the protein product with an N terminus, a hallmark of heliorhodopsin, extended toward the cytoplasm (Fig. 2B). This unique inverse topology suggests that other functions may exist based on accumulated bioinformatics information and the phylogenetic tree of their related gene loci. TfHeR had a relatively long N-terminal region with a relatively large distribution of charged amino acids. Moreover, we showed that there were many negatively or positively charged amino acids forming three loops in the cytoplasmic section (Fig. 2B). Light-driven proton transport assays through photocurrent measurements confirmed that there is no ion-pumping activity (Fig. S3). The p*K*a value of TfHeR was 4.32, determined using pH titration (Fig. S2).

Phylogenetic analysis of TfHeR was performed with branch clusters in phylogenetic trees through alignments involving various microbial rhodopsins (5). TfHeR was classified in the HeR branch community (Fig. 2A). According to a reported study, subclades for various HeR were classified, and TfHeR was included in the classified branch clusters in phylogenetic trees (Fig. S4) (12). Most HeRs are found in Gram-negative bacteria (2). The presence of HeR in Gram-positive bacteria was also reported through sequencing (12). In this study, we managed to obtain HeR from Gram-positive bacteria, proving that HeR is also found in Gram-positive bacteria and wide microbes without distinction.

### Genes on the same operon as HeR.

Based on the phylogenetic tree, we investigated the interacting candidate proteins that might be related to the helio-opsin coding gene of *Trichococcus*. There is a fundamental relationship between gene expression and length, as well as the number and sequence of genes in operons (13). Therefore, we assumed that there was a relationship between the genes on the same operon. The schematic representation of the genes on the operon is shown in Fig. 2A, and we predicted two candidates for interaction with HeR on the same operon in the *T. flocculiformis* genome. These were DUF2238, a protein-coding gene with an unknown function (GenBank accession number WP_086990809.1) and deoxyribodipyrimidine photolyase gene (GenBank accession number WP_086990819.1). The function of DUF2238 was unknown and its interaction with HeR was not clearly defined. Therefore, the *T. flocculiformis* deoxyribodipyrimidine photolyase was selected as a candidate. It is classified as a class 2 cyclobutane pyrimidine dimers (CPD) photolyase, which is mainly found in plants (14). The 12 genera of bacteria with HeR-containing photolyase operons were analyzed using the National Center for Biotechnology Information database (NCBI) (Fig. S1B). Among them, it was observed that subclasses of *Trichococcus* showed the same genomic arrangement (Fig. 2A). Also, the class 2 CPD photolyase found in *T. flocculiformis* was named *T. flocculiformis* photolyase (TfPHR). TfPHR is a class 2 CPD photolyase, which is mainly found in plants. *Trichococcus* species have been isolated from diverse and geographically wide ecosystems. They are found in various polluted geographies like waste treatment systems, swamps, and soils (15). They coexist with plants in this geography, and some genes are transferred through horizontal evolution. Therefore, *Trichococcus* species have genetic constructions, including photolyase in the same operon.

Living organisms suffer from DNA damage caused by UV light. UV-induced dipyrimidine photoproducts, such as CPDs cause mutations due to incorrect genetic information (16). Photolyases can repair the UV-induced DNA lesions activated by blue light. The DNA repair mechanism under only blue light is a momentary reaction, which is the limit of the repair function for DNA damage. FAD is an important cofactor for photolyase redox processes. In a catalytically active and fully reduced form, FAD absorbs visible light in the blue and near-UV range (17). The oxidized photolyase has a yellow color, and the reduced photolyase has a black color (18). The redox state was confirmed using UV-Vis spectroscopy, and the experiment was carried out in a reduced state (17).

### Interaction between TfHeR and CPDII photolyase.

**FIG 3:**
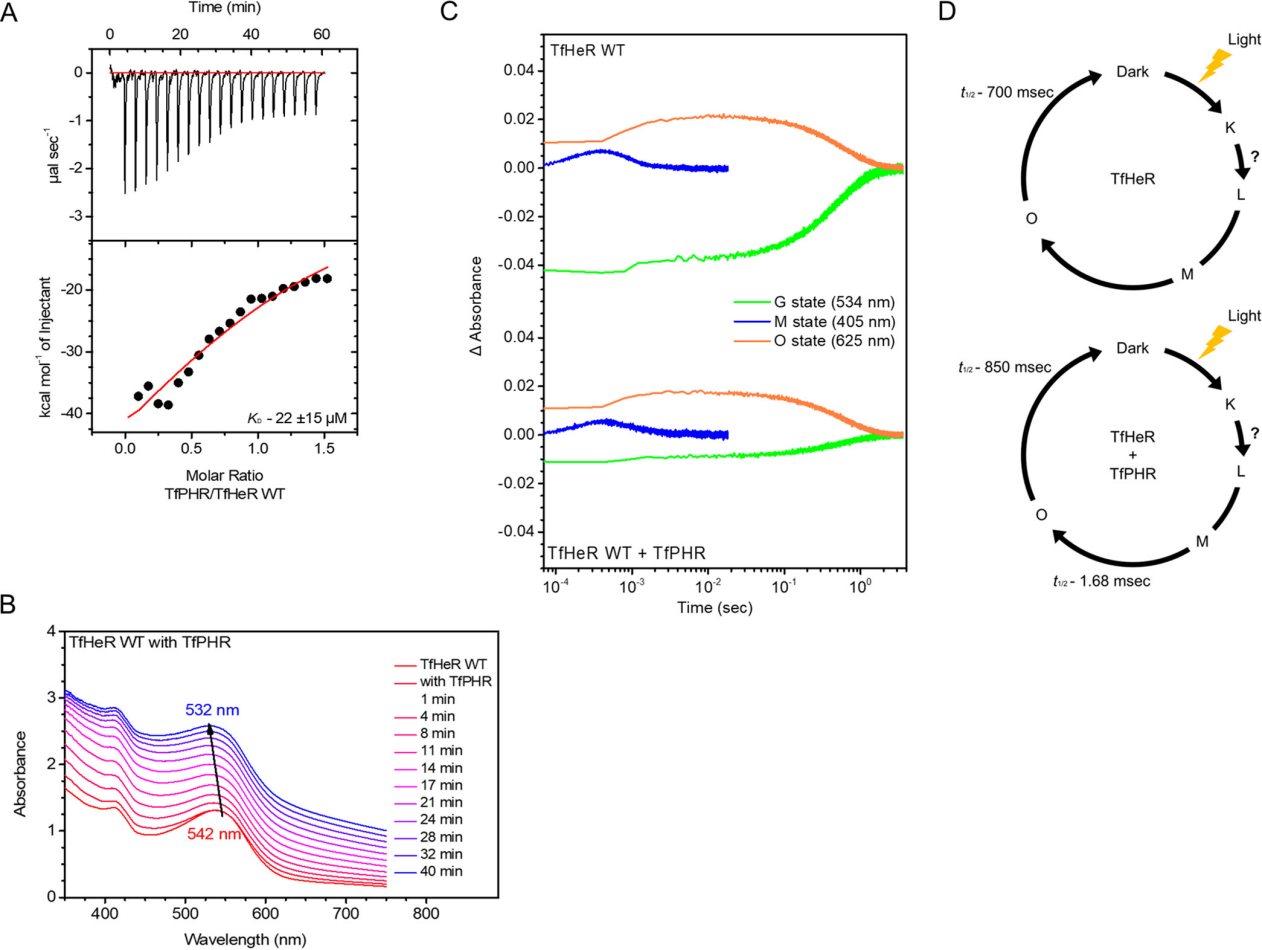
TfHeR interacts with TfPHR. (A) Isothermal titration calorimetry shows the binding results of TfHeR and TfPHR. Top panel, raw data; bottom panel, enthalpy changes in dots per mol. The binding molar ratio of rhodopsin and photolyase is also shown. (B) Absorption spectra of TfHeR were measured when TfPHR was added (1: 0.4 molar ratio, respectively). (C) Transient absorption changed at 544 nm for the Ground-state (green), 405 nm for M intermediate decay (blue), and 625 nm for O intermediate decay (orange). D. Photocycle for TfHeR alone and TfHeR+TfPHR was calculated for each intermediate in each sample.

ITC was previously reported using microbial rhodopsin and transducer (19). Here, a protein-binding assay was performed using isothermal titration calorimetry (ITC) to confirm the interaction between TfHeR and TfPHR (Fig. 3A). In the case of TfHeR and TfPHR, the dissociation constant (*K*_D_) was 22 ± 15 μM, and the ideal S-shaped curve indicated the interaction between the two target proteins (Fig. 3A). Thermodynamic binding parameters of the two proteins were as follows: Gibbs free energy (ΔG) = −53.3 (kJ mol^−1^); enthalpy (ΔH) = −78.8 (kJ mol^−1^); entropy (ΔS) = −1.02 (kJ mol^−1^ K^−1^). *K*_D_ values of sensory rhodopsin and transducer were previously reported as 12.6 μM with ΔG = −28.9 (kJ mol^−1^) (20). *K*_D_ values of TfHeR we identified here were close to those of sensory rhodopsin and the transducer. However, ΔG was lower for TfHeR than that for the binding of sensory rhodopsin and transducer, which is a more favorable reaction to form a stable complex. The *K*_D_ of NpHtrII binding to NpSRII was reported to be 0.65 μM when measured at the same sodium chloride concentration as in the current study. Here, we showed a difference of *K*_D_ value about 36-fold. This suggested that the difference between the binding of a soluble protein and a membrane protein, not the binding of two membrane proteins, might be different due to other factors such as lipids. Spectroscopic analysis showed a blue shift in the absorption spectra due to protein binding (Fig. 3B). The time-dependent spectral shift observed for TfHeR after binding with TfPHR was λ_max_ = 542 nm. It showed an 8 nm shift to 534 nm after 40 min. The spectral shift suggested that TfHeR influenced the chromophore due to the structural change caused by the relatively strong binding with TfPHR with a reasonable *K*_D_ (21, 22). In addition, this binding influenced the chromophore, which possibly changed the photocycle.

Flash laser-induced photocycle measurements were used to compare the photocycle of TfHeR with or without TfPHR (Fig. 3C). The blue-shifted M intermediate accumulation was measured at 405 nm, the red-shifted O intermediate was measured at 625 nm, and the ground state was measured at 544 nm (23). HeR has a long photocycle as previously reported (2). TfHeR also showed a long photocycle-like characteristic. The t_1/2_ values at each state were obtained and compared using multiexponential fitting. As measured for TfHeR, t_1/2_ for the return to the ground state was 0.77 s, that for O decay was 0.7 s, and that for M decay was 1.18 ms. A slow photocycle was observed when TfHeR was bound to TfPHR. For the photocycle of TfHeR bound to TfPHR, G recovery slowed to 0.85 s and M decay slowed to 1.68 ms. O decay was measured as 0.85 s, which was slower than that of TfHeR alone. This suggested that TfPHR and TfHeR interactions might influence proton recovery to the Schiff base and consequently affect recovery to the G state. The 48C12 HeR functions as a proton acceptor in the water cluster of the Schiff base cavity [SBC] (12). Protein binding might influence the conformational change from the M to the O state, which may delay the proton movement in the accepting group. Two photocycle schemes for TfHeR bound to TfPHR were obtained (Fig. 3D).

### *In vivo* and *in vitro* analyses for the regulation of CPDII photolyase by TfHeR.

Since the photocycle was influenced by the binding between TfHeR and TfPHR, the binding might also influence their function. Photolyase is regulated by microbial rhodopsin that its function is regulated by light. It is photoexcited under visible light, and CPD splitting is handled by FADH^−^. We performed *in vitro* and *in vivo* experiments on DNA repair by photolyase (Fig. 1B and C). *In vitro* experiments were performed to investigate the DNA repair efficiency of TfPHR in the presence and absence of TfHeR (Fig. 1B). In addition, the effect of green light, which is not the activating light for photolyase, was used as a control. The change in the absorbance of recovered UV-damaged oligonucleotide dT (20 mer) was measured using white light irradiation at 265 nm (Fig. 1B top) (24). As a result, the repair activity of TfPHR in the presence of TfHeR was approximately three times higher than that of TfPHR alone based on the saturation state. In addition, the comparison of DNA repair efficiencies using only blue light (430 nm) showed similar results in both cases (Fig. 1B bottom). Another experiment was performed by irradiating TfHeR using green light (532 nm). DNA repair was not observed with TfPHR in the presence of green light. However, DNA damage was repaired when TfPHR was bound to TfHeR under green light (Fig. 1B, middle). To confirm the effect of negative control in the green light exposure experiment, a DNA repair experiment was performed with Gloeobacter-rhodopsin and TfPHR, but there was no influence on the repair activity. However, as is shown in the following results, we revealed R162 and K227 as sites that affect the binding of TfHeR and TfPHR. Therefore, we wanted to confirm whether the function of TfPHR is affected by mutations on these sites. TfHeR R162Q and K227Q mutants did not affect the function of TfPHR under white, blue, and green light. This suggested that TfHeR binding with TfPHR might be involved in electron transfer to allow the formation of photoexcited TfPHR with a conformational change in TfHeR. These results indicated the increased efficiency of the photolyase in DNA repair through the electron transfer mechanism. We suggested that TfHeR might broaden the spectrum at which the photolyase is active so that the photolyase would absorb green light. These results suggested that the activity spectrum of TfPHR might be evolutionarily expanded by TfHeR, which has a unique action spectrum.

*In vivo* experiments were conducted to investigate the function of TfPHR bound with TfHeR in live cells. Cell survival upon UV damage was monitored using E. coli K-12 derivative JW0698, which is a photolyase-knockout strain (*phr*-) (25). The plasmids carrying TfHeR- and TfPHR-coding genes were transformed into *phr*- cells. The results of *in vivo* experiments were consistent with those of *in vitro* experiments. In the case of *phr*- cells, cell survival was abrupt due to the absence of photolyase under white light. However, cells expressing TfPHR showed better survival. The survival of the cells expressing TfHeR+TfPHR was higher than that of the *phr*- cells, with the *phr*- cells expressing TfPHR upon UV damage (Fig. 1C, top). These results were consistent with the *in vitro* results. In addition, the trend observed under blue light was similar to that observed under white light and *in vitro* experiments (Fig. 1C bottom). The survival of cells expressing TfPHR+TfHeR showed a less steep slope than that of *phr*- cells, *phr*- cells expressing TfPHR under blue light. An interesting trend was observed under green light, which was similar to the *in vitro* results (Fig. 1C middle). TfPHR alone gave a similar result to the photolyase-knockout experiment. However, the survival of the cells treated with TfHeR+TfPHR was higher than that of *phr*- cells, *phr*- cells expressing TfPHR. In addition, the R162Q and K227Q mutants of TfHeR gave results consistent with the *in vitro* results. Since it is difficult for the two mutants to bind to TfPHR, it was confirmed whether the function of TfPHR upon UV damage was affected by the mutations. TfHeR R162Q and K227Q mutants did not affect the function of TfPHR under white, blue, and green light. It caused a decreased survival of the cells compared to wild-type TfHeR. This tendency was consistent with the results of *in vitro* experiments, where the effect of TfPHR binding to TfHeR was relatively lower under green light than that under white light. These results suggested that TfHeR might act as a regulator that functions by inducing a photoexcited state via electron transfer from TfHeR within the broad wavelength region.

### Critical residues in the loop of TfHeR for CPDII photolyase biding.

**FIG 4:**
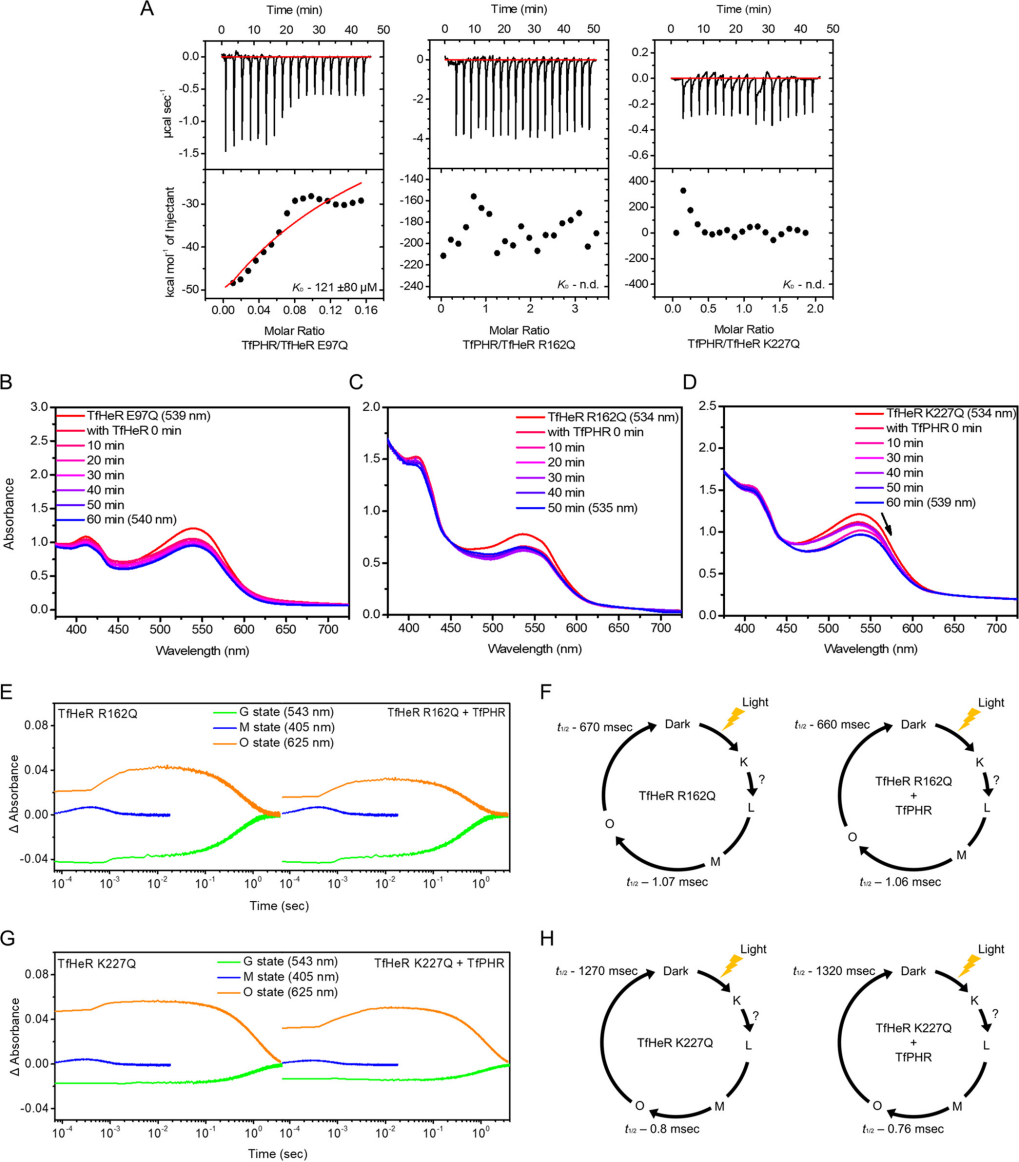
Analysis of TfHeR mutants. (A) ITC shows the binding of TfHeR-E97Q to TfPHR (left). Top panel, raw data; bottom panel, enthalpy change. Figures in the middle and right show the binding of TfHeR-R162Q and TfHeR-K227Q to TfPHR, respectively. n.d, not determined. (B–D) The spectral shift for R162 and K227. For the wild type, a blue shift was observed. TfPHR:mutant molar ratio was 1:4. A 4 nm blue shift was observed for the E97Q mutant. For R162Q, there was no shift for 50 min. For K227Q, a 5 nm red shift was observed after 60 min. (E and G) Transient absorption changed at 544 nm for the Ground-state (green), 405 nm for M intermediate decay (blue), and 625 nm for O intermediate decay (orange) for R162 and K227. (F and H) Photocycle for R162 and K227 alone and TfHeR+TfPHR was calculated for each intermediate in each sample.

Based on the above results that indicated TfHeR binding modified TfPHR activity, we started to look for specific residues for interaction. We focused on the loops in which the binding region was located, corresponding to the cytoplasmic side of TfHeR and identified three amino acid residues using several mutants. E97, R162, and K227 were selected, and ITC was performed to determine whether residues affected binding (Fig. 4A). R162 and K227 were replaced with glutamine to remove the charge affinity. We showed that mutations on R162 and K227 resulted in no TfPHR binding. This meant that the positive charge had a critical influence on the binding between TfHeR and TfPHR. However, in the case of the E97Q mutant, *K*_D_ value was 121 ± 80 μM, which was approximately six times lower than that of the wild type. The thermodynamic parameters of binding were weak with ΔG = −4.66 kJ mol^−1^. These results suggested that the E97 position might enhance the interaction. In contrast, we identified that R162 and K227 were amino acid residues essential for binding, and E97 played an important role in the binding affinity between TfPHR and TfHeR.

Spectroscopy was performed using a mixture of TfPHR to confirm that each residue was important for binding (Fig. 4B to D). TfPHR was incubated with the E97Q mutant, and the spectra were blue-shifted by only 4 nm. It showed a small shift compared to the 8 nm blue shift measured for the wild type. It was consistent with the ITC data that indicated low binding affinity, suggesting that E97 enhanced the binding affinity between TfHeR and TfPHR. The R162Q mutant maintained maximum absorption without spectral shift, indicating that R162 was essential for binding. However, in the case of K227Q, the absorption spectra were red-shifted by 5 nm. These could be the result of spectral shifts caused by incorrect structural binding because the R162 site was maintained in the K227Q mutant. However, in the case of R162Q, there was no shift, which indicated a more critical site for binding.

The t1/2 values in each state were obtained and compared using multiexponential fitting. For TfHeR R162Q, t1/2 for the return to the ground state was 0.72 s, that of O decay was 0.67 s, and that of M decay was 1.07 ms. It showed a similar photocycle to the wild-type TfHeR. For the photocycle of TfHeR R162Q bound to TfPHR, G recovery slowed to 0.7 s and M decay slowed to 1.06 ms. O decay was 0.66 s, which was similar to the TfHeR R162Q mutant. In the case of TfHeR R227Q, t1/2 for the return to the ground state was 1.08 s, that of O decay was 1.27 s, and that of M decay was 0.8 ms. For the photocycle of TfHeR K227Q bound to TfPHR, G recovery was measured as 1.12 s and M decay as 0.76 ms. O decay was 1.32 s, which was similar to the TfHeR K227Q mutant. TfHeR R227Q and K227Q mutants showed photocycles similar to those in the absence of TfPHR. Unlike the O decay, which was slowed upon binding of TfPHR to TfHeR, showed that the mutants do not bind TfPHR. The K227Q mutant showed a relatively slow O decay compared to the wild-type TfHeR. Because it is close to the site that is responsible for maintaining the polar cavity in the cytoplasmic section, it might affect the photocycle due to changes in the cluster of water molecules and the change in polarity (12).

In this study, we reported the function of HeR, a different class of type-1 microbial rhodopsin. Protein interaction was confirmed through ITC, spectroscopy, photocycle analysis, and site-directed mutagenesis. Through *in vivo* and *in vitro* experiments, HeR function was identified in regulating the photolyase. The function of TfHeR was revealed, which is to regulate the UV-induced DNA damage repair by TfPHR. TfHeR expanded the active spectrum of TfPHR to the green region. We showed that TfHeR binding to TfPHR enhanced DNA repair activity under green light, which can reverse UV damage induced under blue light.

### Conclusion.

We report the function of new rhodopsin which is an inverted 7-transmembrane protein, Tfheliorhodopsin (TfHeR), isolated from Trichococcus flocculiformis. The *T. flocculiformis* deoxyribodipyrimidine photolyase was selected and tested for interaction as a candidate. The thermodynamic binding parameters of the two proteins were ΔG = −53.3 (kJ mol^−1^), ΔH = −78.8 (kJ mol^−1^), and ΔS = −1.02 (kJ mol^−1^ K^−1^). The time-dependent spectral shift of TfHeR after mixing with TfPHR was λ_max_ = 542 nm, showing an 8 nm shift to 534 nm after 40 min. The chromophore was influenced due to the structural change caused by the relatively strong binding of the two proteins with a reasonable *K*_D._ Flash laser-induced photocycle measurements were used to compare the photocycle of TfHeR with or without TfPHR. Protein binding may influence the extensive conformational change from the M to the O state, which may delay the proton movement in the accepting group. Based on *in vivo* and *in vitro* experiments, we showed the binding of heliorhodopsin improved the function of photolyase by about 3-fold to repair UV-caused DNA damage. Also, the enhanced DNA repair of TfHeR/TfPHR was observed in the presence of green light. Our results suggested that rhodopsin directly controls photolyase activity and coevolves to broaden the action spectrum via protein-protein interaction. We suggest an evolutionary effort to overcome the functional limit through a mutual relationship of proteins in microorganisms. Next, we would like to study the electron transfer mechanism in photochemical reactions. The relationship between the photochemistry of heliorhodopsin and the electron transfer mechanism of the photolyase will be further studied.

## MATERIALS AND METHODS

### Cloning.

Trichococcus flocculiformis KACC 14619 was obtained from the Korean Agricultural Culture Collection (KACC). *T. flocculiformis* heliorhodopsin (TfHeR; accession no. WP_086990819.1), and *T. flocculiformis* photolyase (TfPHR, accession no. WP_086990817.1) genes were obtained through genomic DNA PCR (PCR) using specific primers. Forward and reverse primers containing a hexahistidine tag at the C terminus for TfHeR were 5′-CATATGAAAGAGATCACGACAGAAAAA-3′ and 5′-GCGGCCGCTTAGTGATGATGGTGGTGATGCGGCTGCATC-3′, respectively. Forward and reverse primers containing a hexahistidine tag at the C terminus for TfPHR were 5′-CATATGATACTGGAAGAAAGAATC-3′ and 5′- GCGGCCGCTTAGTGATGATGGTGGTGATGGTTGCGTTTGCG-3′, respectively. The PCR fragments were introduced between *Nde I* and *Not I* restriction enzyme sites in the pKA001 vector (26).

### Protein analysis and phylogenetic tree.

The similarity of the amino acid sequence of HeR 48C12 to the data stored in the NCBI database was determined using BLAST-P. Multiple sequence alignments were constructed using MUSCLE (27). The transmembrane TfHeR 2D structure was predicted using Philius (28). The operons for several genomes of each bacterium containing photolyase were investigated using the NCBI GenBank database. To visually show the location of type-1 rhodopsin, HeR, and TfHeR, we used an evolutionary analysis of the phylogenetic tree using the maximum likelihood method. The evolutionary history was inferred using the maximum likelihood method and a JTT matrix-based model (29). Evolutionary analyses were conducted using MEGA-X software (30).

### Protein expression in E. coli UT5600.

pKA001-TfHeR and pKA001-TfPHR were transformed into E. coli UT5600, and single colonies were selected and grew in Luria-Bertani (LB) medium containing 50 μg/mL ampicillin at 35°C, shaking at 200 rpm overnight. After the overnight culture, 1% of the total volume was transferred and grown to an optical density (OD) of 0.4 at 600 nm. Expression was induced by adding 1 mM isopropyl β-D-1-thiogalactopyranoside (IPTG; Duchefa Biochemie) and 7 μM all-transretinal (Sigma) for 4 h at 35°C, shaking at 200 rpm. TfPHR expression was induced only with IPTG. The cells were harvested via centrifugation for 15 min at 5,000 rpm and 4°C, and subsequently, washed two times (Eppendorf centrifuge 5810R) for 20 min at 4,000 rpm and 4°C with sonication buffer (150 mM NaCl, 50 mM Tris-HCl at pH 7.0).

### Purification of TfHeR and TfPHR.

The harvested cells containing TfHeR were lysed via sonication and ultracentrifuged for 1 h at 35,000 rpm at 4°C (Beckman ultracentrifuge) at the Advanced Bio-Interface Core Research Facility, and the pellets were resuspended in 1% n-dodecyl-β-D-maltopyranoside (DDM, Anatrace, USA) overnight at 4°C. After solubilization, another centrifugation was performed for 15 min at 20,000 rpm and Ni^2+^ NTA agarose (Qiagen) was added to the supernatant. The mixture was gently shaken for 4 h at 4°C. The expressed rhodopsins were separated using affinity chromatography. Purified rhodopsin was concentrated using an Amicon Ultra-4 10 K centrifugal filter tube (Millipore). In the case of TfPHR, the cells were disrupted via sonication. After removing cell debris through centrifugation, the cell lysate was subjected to Ni^2+^-NTA agarose. TfPHR was eluted with sonication buffer containing 250 mM imidazole and subsequently concentrated using an Amicon Ultra-4 10 K centrifugal filter tube.

### Absorption spectroscopy and determination of p*K*a.

The absorption spectra of the purified rhodopsins were measured at different pH values using a UV-VIS spectrophotometer (Shimadzu UV-2550). Each spectrum was corrected to the baseline spectrum at a pH of 7.0. The wavelengths of the top or bottom peaks from different spectra were selected to calculate the p*K*a. The absorbance for each pH value at different wavelengths was calculated relative to the deprotonated forms, and the p*K*a was fitted using Origin 9.0. The corrected ratio of protonated or deprotonated forms at different pH values was estimated and fitted using functions containing one or two p*K*a components (y = A/[1 + 10pH-p*K*a]).

### Light-driven proton transport assay.

The harvested cells in 500 mL of LB broth were resuspended in 20 mL of buffer A (20% sucrose, 30 mM Tris-HCl at pH 8.0) containing 100 μg/mL lysozyme and stirred gently at room temperature for 30 min. Spheroplasts were collected via centrifugation at 3,000 × *g* at room temperature for 20 min and then resuspended in 800 μL of buffer B (100 mM KPi at pH 7.0, 20 mM MgSO_4_, 20% sucrose, and 8 mg DNase I). Then, they were injected gently using a 1 mL syringe (18- gauge-needle) into 400 mL of buffer C (50 mM KPi at pH 7.0) shaking at 37°C and 200 rpm for 15 min. Exactly 8 mL of 0.5 M Na_2_-EDTA at pH 8.0 was added and the mixture was shaken. After 15 min, 12 mL of 0.5 M MgSO_4_ was added and shaken for 15 min. Membrane vesicles were isolated via ultracentrifugation at 20,000 rpm and 4°C for 30 min and resuspended in 1 mL of buffer D (10 mM MgSO_4_ and 100 mM KPi at pH 7.0). The membrane vesicles were spun via ultracentrifugation at 20,000 rpm and 4°C for 30 min and resuspended in an unbuffered solution (10 mM NaCl, 10 mM MgCl_2_, 100 μM CaCl_2_). The pH of membrane vesicles with rhodopsin was adjusted to 8.0 using HCl or NaOH and illuminated at 100 W/m^2^ using a short wave 500 nm cutoff filter (Sigma Koki SCF-50S-50Y) in combination with wide-band interference and heat-protection (2.5% CuSO_4_·5H_2_O and concave lens for collecting light) filters. This assay was performed in the presence or absence of 10 μM carbonyl cyanide m-chlorophenylhydrazone (CCCP). The pH value was monitored using a pH meter (Horiba pH meter F-51) with a pH electrode bar (Horiba pH electrode bar 9618S-10D). The pH data were recorded automatically using the Horiba data Navi software. The difference in the protons was calculated and fitted with pH = −log[H+].

### Measurement of TfHeR and TfPHR binding affinity using ITC.

For ITC analysis (20), wild-type and mutant TfHeR along with TfPHR were completely replaced with sonication buffer containing 0.02% DDM using Amicon Ultra-4 10,000 MWCO centrifugal filter units. ITC analysis was performed using a MicroCal ITC200 instrument (Malvern Panalytical). Data analysis was performed using the Origin-ITC software.

### TfPHR enzyme assay using oligonucleotide (dT)_20_.

The synthesized 20 mer oligonucleotide (dT) (Cosmogenetech, South Korea) was irradiated overnight at room temperature at 253 nm. The purified photolyase was concentrated and replaced with reaction buffer (50 mM Tris-HCl at pH 7.0, 1 mM Na_2_-EDTA, 100 mM NaCl, 5% glycerol, and 14 mM dithiothreitol) (24). Oxygen was degassed using N_2_, and subsequently, the CPD oligonucleotide (dT) was added at a final concentration of 5 μM in a final volume of 8 mL. In the dark, TfHeR and TfPHR were added at a final concentration of 50 nM and activity was measured every 5 min. The enzyme activity was terminated at 80°C, and white, green LED (532 nm), and blue LED (430 nm) lights were applied. Another measurement was performed at 265 nm using a NanoDrop 2000/2000c Spectrophotometer (Thermo Scientific) for each sample after the termination of enzyme activity.

### Cell viability assay.

E. coli cell viability assay was performed as described in a previous study (31). However, some modifications were made according to the experimental conditions. This assay was performed using a *phr*-deficient strain, E. coli JW0698 (25). TfHeR- and TfPHR-coding genes were cloned into pKA001, which is regulated by the *lac* and *araBAD* promoters. These plasmids were transformed into E. coli JW0698 cells. The transformed cells were incubated and induced with 0.84 mM IPTG and 0.2% l-arabinose. Serial dilutions of each transformation were plated on agar plates with LB alone (for JW0698) and LB containing ampicillin (for JW0698/TfPHR and JW0698/TfPHR+TfHeR). LB agar plates were irradiated with UVC light. The white, green LED, and blue LED lights were applied for 1 h after UVC light irradiation to induce the DNA repair process against UV damage. The exposed plates were incubated overnight at 37°C, and the number of colonies was counted to quantify survival.

### The spectral shift upon TfHeR binding to TfPHR.

The purified TfHeR and TfPHR were mixed at a 1:4 molar ratio, and the time-dependent absorption spectra were measured using a UV-2450 spectrometer.

### Flash laser-induced photocycle measurements.

The flash-induced absorption transients were measured using the flash photolysis system designed by Yang et al. (23). TfHeR and TfPHR were prepared in a sonication buffer containing 0.02% DDM. The photocycle of TfHeR was measured as 1 OD measurement of protein, and the change in transient absorbance was measured at room temperature. The measured data, which were averaged, indicated the decay and formation of the intermediates using the activation of a specific green laser (532 nm).

### Light-driven photocurrent measurements.

Photocurrent measurements based on the protein current under light illumination were designed according to previous studies (23, 32). Protein photocurrent was measured in a photochemical cell with a green laser (532 nm). From the top of the cassette, it consisted of an ITO-coated glass slide, a sample chamber, a dialysis membrane, a blank chamber, and an ITO-coated slide, which was connected to a wire and a signal amplifier SR570 (Stanford Research Systems). The samples were measured in an unbuffered solution, and the data were measured 64 times. The values were averaged.

### Data availability.

All data are available in the main text or the supplemental materials.
