# Supplementary material for: Heliorhodopsin Helps Photolyase to Enhance the DNA Repair Capacity
Source: Microbiol Spectr. 2022 Oct 11;10(6):e02215-22. doi: 10.1128/spectrum.02215-22 (PMC9769723; doi:10.1128/spectrum.02215-22)
Supplement: Supplemental file 1 — Supplemental material. Download spectrum.02215-22-s0001.pdf, PDF file, 1.4 MB [file spectrum.02215-22-s0001.pdf]

Supplemental Information for Heliorhodopsin helps photolyase to enhance the DNA repair capacity and broaden its spectrum

Jin-gon Shim, Shin-Gyu Cho, *Se-Hwan Kim,* Kimleng Chuon, Seanghun Meas, *Ahreum Choi, Kwang-Hwan Jung* [a,*]

[a] *Department of Life Science and Institute of Biological Interfaces, Sogang University, Seoul, South Korea*

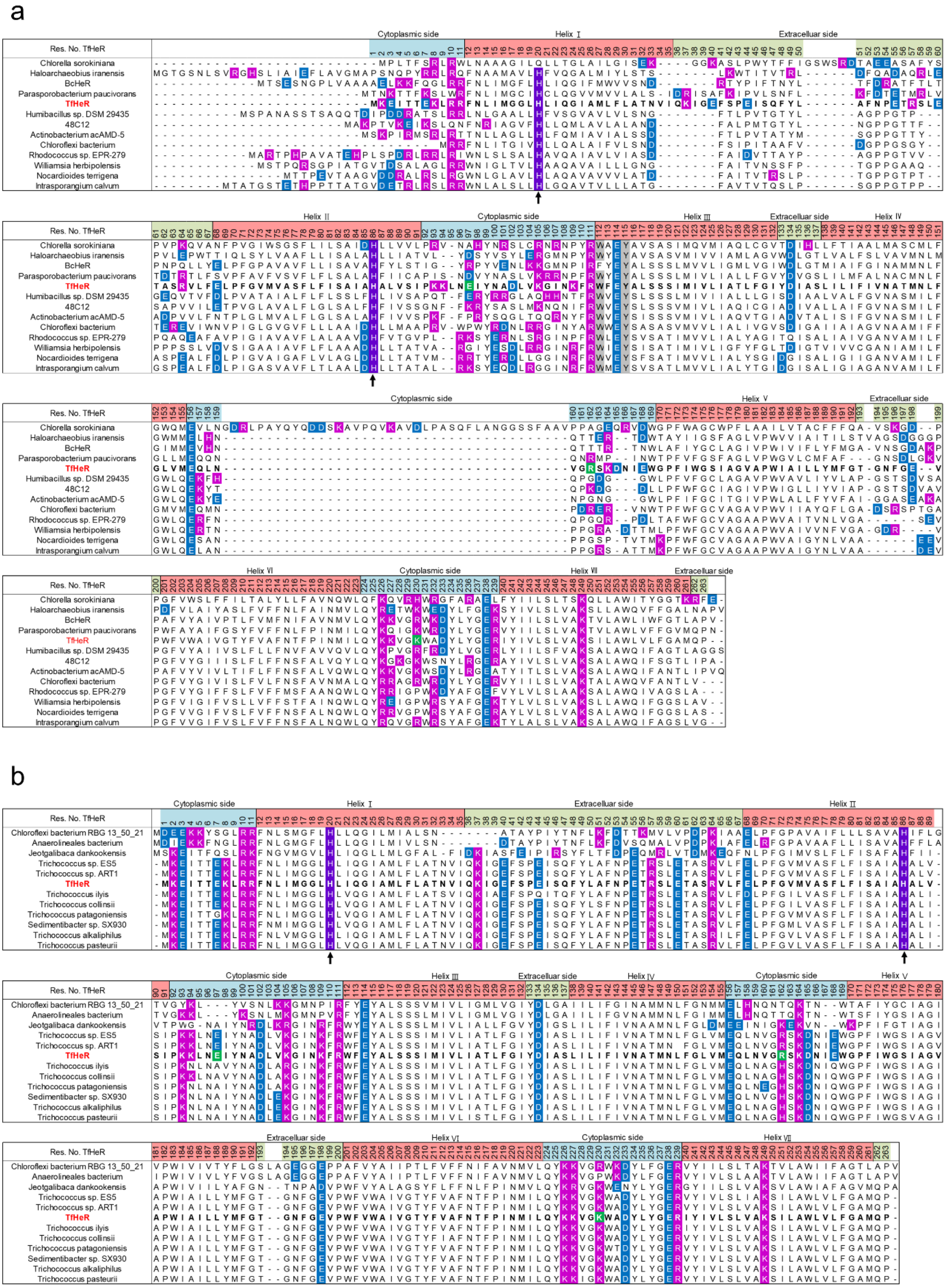

Supplementary Figure S1 | Alignment of the amino acid sequence of TfHeR with that of heliorhodopsin. Transmembranes and multiple alignments of heliorhodopsins were visually

designated. In the secondary transmembrane, each helix is in red; cytoplasmic side is in light blue; extracellular side is in light green. In the specific residues, negatively charged residues are in blue; positively charged residues are in pink; specific residues of TfHeR are in green. Proton-accepting groups are marked with black arrows and are highlighted in purple. **a.** Multiple alignments of the amino acid sequence of heliorhodopsin with that of predicted and published rhodopsin. **b.** Multiple amino acid sequence alignments of heliorhodopsin-containing photolyase operons. NCBI reference numbers are as follows: *Chloroflexi* bacterium RBG 13_50_21, OGN94913.1; *Anaerolineales* bacterium, WP_116779633.1; *Jeotgalibaca dankookensis*, WP_062469668.1; *Trichococcus* sp. ES5, WP_068562519.1, *and Trichococcus* sp. ART1, WP_119094144.1; *Trichococcus ilyis*, WP_068623315.1; *Trichococcus collinsii*, WP_086985642.1; *Trichococcus patagoniensis*, WP_108032300.1; *Sedimentibacter* sp. SX930, WP_086628472.1; *Trichococcus alkaliphilus*, WP_106449223.1; *Trichococcus pasteurii*, WP_086943172.1

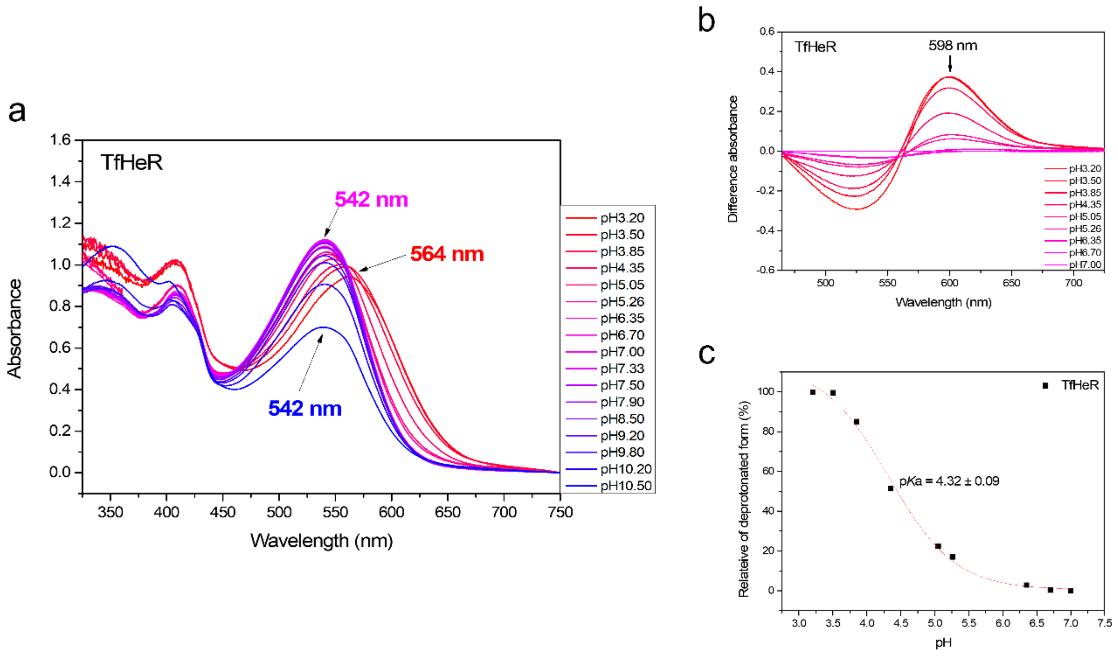

Supplementary Figure S2 | Absorption spectrum of TfHeR WT at different pH values. a. The experiment was conducted under acidic conditions with decreased pH and alkaline conditions with elevated pH from pH 7. In acidic conditions, it was red-shifted from 562 nm to 564 nm. Under alkaline conditions, there was no shift from 542 nm to 542 nm. **b.** The spectrum of different pH values was subtracted based on pH 7.0, and the p*K*a was determined by selecting the absorption at 598 nm from the different absorption spectra. **c.** Estimated p*K*a values relative to the deprotonated forms determined via spectroscopic pH titration. The p*K*a of the counterion was estimated to be 4.32

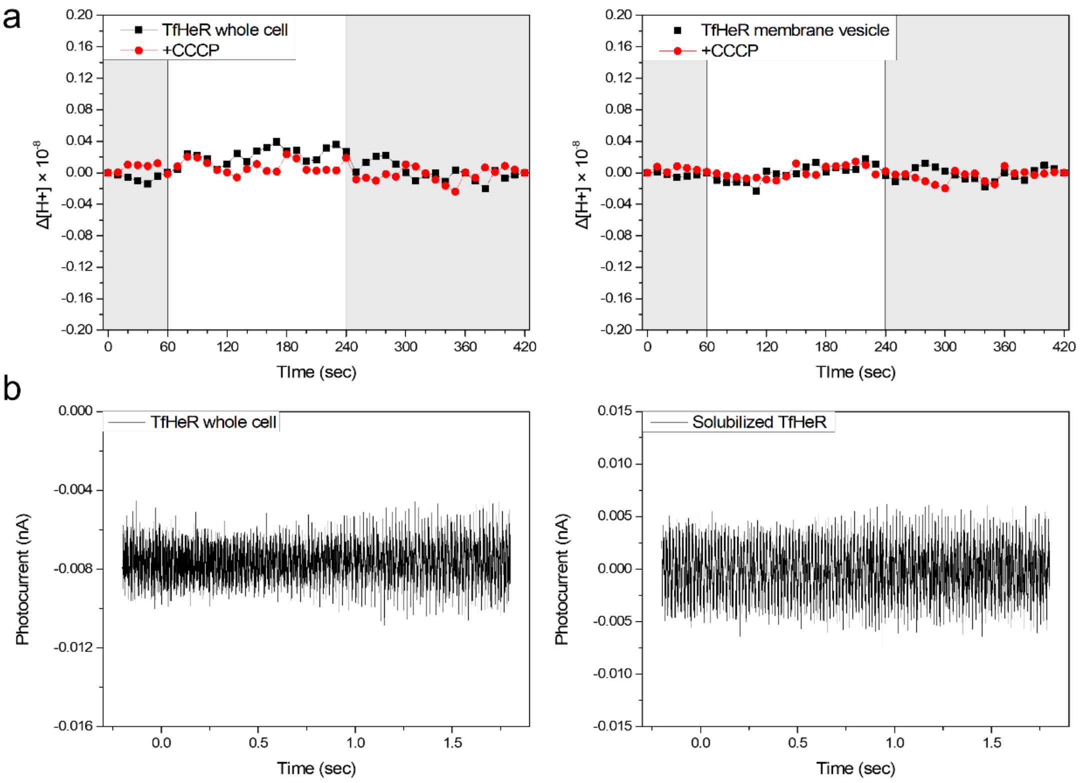

Supplementary Figure S3 | Light-driven proton transport assays and photocurrent measurements. To determine the properties of WT TfHeR, proton transport assay was performed. **a.** Light-driven proton transport assay using two cell types. Figures on the top left and right show measurements using whole cell and membrane vesicles in an unbuffered solution (10 mM NaCl, 10 mM $MgCl_2$, and 100 µM $CaCl_2$) using a pH meter, respectively. The black and red dotted lines indicate the absence and presence of 10 µM CCCP, respectively. Both experiments were conducted under the same conditions, and dark and light adaptation are indicated with grey and white areas, respectively. **b**. Photocurrent was measured using whole cells and purified proteins. It is confirmed that the proton movement by irradiating the ITO-coated glass chamber under green light (532 nm). In both cases, no proton movement was observed. Measurements are an average of 64 experiments.
